# Homeobox genes gain trimethylation of histone H3 lysine 4 in glioblastoma tissue

**DOI:** 10.1042/BSR20160028

**Published:** 2016-06-17

**Authors:** Kun Luo, Donghui Luo, Hao Wen

**Affiliations:** * Department of Neurosurgery, Xinjiang Evidence-Based Medicine Research Institute, First Affiliated Hospital of Xinjiang Medical University, Urumqi 830054, China; † Department of Neurology, First Affiliated Hospital of Xinjiang Medical University, Urumqi 830054, China; ‡ State Key Laboratory Incubation Base of Xinjiang Major Diseases Research, First Affiliated Hospital of Xinjiang Medical University, Urumqi 830054, China

**Keywords:** glioblastoma multiforme (GBM), H3K4me3, homeobox genes

## Abstract

Glioblastoma multiforme (GBM) exhibits considerable heterogeneity and associates with genome-wide alterations of the repressed chromatin marks DNA methylation and H3 lysine 27 trimethylation (H3K27me3). Tri-methylation on lysine 4 of histone H3 (H3K4me3) is an activating epigenetic mark that is enriched at promoter and promotes expression. It will be helpful in GBM diagnosis and treatment to identify the alteration of H3K4me3 between human GBM and GBM-surrounding tissues. Here, we performed an analysis using next-generation sequencing techniques to identify H3K4me3 modification in a case of GBM and the GBM-surrounding tissues. The results revealed a global decrease in H3K4me3 in GBM, especially at promoters and CpG islands. In GBM, homeobox genes gain H3K4me3, whereas the cell–cell adhesion-related cadherin genes lose H3K4me3. The products of the homeobox genes are highly connected with Ras-signalling and PI3K-Akt signalling pathways. Using The Cancer Genome Atlas (TCGA) data, we inferred the homeobox-regulated genes’ expression is higher in 548 GBM cases than in 27 lower grade glioma cases giving that OLIG2 expression can be a reference. The results suggested that the H3K4me3 alteration is related to the formation and migration of GBM cells. We also found an extremely high reads count at epidermal growth factor receptor (EGFR) promoter, probably due to an amplification of copy number. Our analysis provides a case study about the change of H3K4me3 during shift to GBM.

## Abstract (precis)

We revealed that in GBM homeobox genes gain tri-methylation on lysine 4 of histone H3 (H3K4me3), whereas the cell–cell adhesion-related cadherin genes lose H3K4me3, suggesting that the H3K4me3 alteration is related to the formation and migration of GBM cells.

## INTRODUCTION

Glioblastoma multiforme (GBM) is an aggressive and lethal cancer. Sturm et al. [1] suggested six subgroups for GBM: isocitrate dehydrogenase (IDH), K27, G34, RTK I and II, and MES [1]. The first three subgroups are associated with mutations IDH1 (R132H), H3F3A (K27) and H3F3A (G34); and the subgroups RTK I and II are associated with platelet-derived growth factor receptor (PDGFRA) and epidermal growth factor receptor (EGFR) amplifications respectively and both with CDKN2A deletion [1].

Besides genetic abnormity, aberrant epigenetic alteration also contributes to GBM [1,2]. IDH, K27 and G34 subgroups show alterations in both DNA methylation and tri-methylation on H3K27 (H3K27me3). IDH1 subgroup displays hypermethylation, whereas H3F3A (G34) subgroup shows hypomethylation [3,4]. Global reductions of H3K27me2 and H3K27me3 were found in H3F3A (K27M) subgroup [4]. In IDH1-mutated cells, it was thought that overproduction of 2-hydroxyglutarate inhibits the TET family of 5-methylcytosine hydroxylases and H3K27-specific demethylases, thus leading to a decrease in 5-hydroxymethylcytosine and an increase in H3K27 methylation [5].

H3K4me3 is generally associated with active chromatin and marks at promoters of expressed genes [6]. In GBM, alterations in DNA methylation are highly associated with changes of H3K4me3. In high CpG density promoters, loss of H3K4me3 and retention of H3K4me2 or H3K27me3 are correlated with an increase in DNA methylation [7]. H3K4me3 shows a complicated alteration in GBM. It increases in some GBM cases, but reduces in other cases. Recently, the combinatorial loss of both H3K4me3 and H3K27me3 was identified in majority of paediatric GBMs with the mutations in H3.3-ATRX-DAXX pathway [8]. In primary GBM, it was found that the recurrent hypomethylation of TERT, which encodes telomerase reverse transcriptase, and oncogenes GLI3 and TP73, resulting in increases of H3K4me3 and transcription [9]. Elevated levels of H3K4me3 were also observed in MRI-classified GBM of the subventricular zone in *Papio anubis* [10]. The H3K4 methyltransferases, such as MLL and SMYD3, were found closely associated with GBM [11,12]. MLL can directly activate the homeobox gene HOXA10 and contributes to the tumorigenic potential of glioblastoma stem cells [13]. Overexpression of SMYD3 was found associated with glioma tumorigenicity through P53 [12].

Here, we reported genome-wide analysis of H3K4me3 in both GBM and GBM-surrounding tissues so as to determine the H3K4me3 alteration between two kinds of tissues and resolve the biological meaning of the alteration. DNA methylation and H3K27me3 have been extensively studied in GBM [1,4]. Although changes in H3K4me3 are reversibly linked to DNA methylation in GBM [7,9], there is still a need to explore the detailed distribution of H3K4me3. Moreover, in the studies of Chan et al. [4] and Schwartzentrube et al. [3], epigenetic comparisons were carried out between GBM and neural stem cells [3,4]. Thus, a direct analysis between the differences in GBM and GBM-surrounding tissues would be interesting because it might provide more detailed information regarding GBM growth.

Our results suggested an H3K4me3 reduction in GBM. Importantly, we found that the homeobox genes gain H3K4me3 modification and the cadherin genes lose the modification. The homeobox proteins connect to cancer-related pathways and the cadherin proteins function in cell–cell adhesion, suggesting that the alteration in H3K4me3 is closely associated with GBM formation and migration. We also inferred the subgroups of GBM with H3K4me3 chromatin immunoprecipitation sequencing (ChIP-Seq) data.

## MATERIALS AND METHODS

### Chromatin immunoprecipitation sequencing (ChIP-Seq) of H3K4me3

We collected a surgically removed specimen of magnetic resonance imaging-identified GBM from a 63-year-old female (Supplementary Figure S1A). In MRI scanning, one mass was found in right frontal lobe and it was identified as glioblastoma multiforme (WHO Grade IV) in regular pathology. Immunohistochemistry indicated GFAP+, OLIG2+, EMA+, VIM+, NEU-N+ and CD34+. We firstly discriminated the GBM from GBM-surrounding tissue according to colour, quality and blood supply. Secondly, the GBM and GBM-surrounding tissue were histologically confirmed according to WHO classification of tumours of central nervous system. At last, the confirmed GBM and GBM-surrounding tissue were collected. We provided two images of the immunohistochemistry of OLIG2 and GFAP for the sample (Supplementary Figures S1B–S1C). GBM and GBM-surrounding tissues were separated according to the guide of the immunohistochemistry technique. The use of human specimens was approved by the First Affiliated Hospital of Xinjiang Medical University. The tissues were stored at −80°C. The GBM-surrounding tissue is used as a control in the study.

A ChIP kit (Product ID: 53040) was purchased from Active Motif. ChIP experiments were carried out according to the manufacturer's instructions. An anti-H3K4me3 antibody (Product ID: 17-614) was purchased from Merck Millipore. DNA was extracted using a Gel Extraction kit (Qiagen) (Supplementary Figure S1D). A DNA library was prepared and sequenced using an Illumina Genome Analyzer II (Illumina) according to the manufacturer's instructions.

### Normalized reads counts and profiles near specific sites

Raw sequencing reads were mapped on to the human genome (hg19) using Bowtie [14]. Only uniquely mapped reads were used for further analysis. Firstly, Watson- and Crick-strand reads were shifted by 50 bp in the 5′ direction. The absolute read counts of each genomic site were expressed as the number of reads covering the genomic sites. Secondly, the read counts were normalized by dividing the values by the average read counts of the whole genome.

Genomic coordinates of transcription start sites (TSSs), transcription termination sites (TTSs), CpG islands, and conserved transcription factor-binding sites (TFBSs) were retrieved from the UCSC genome browser using the tables function for version hg19 (http://genome.ucsc.edu) [15]. H3K4me3 profiles near specific sites, e.g. TSSs, were the average profile by summing the normalized read counts at each genomic site and then dividing the summated signal by the gene number.

### Identification of H3K4me3 peaks

The H3K4me3 peaks were identified by ‘broad’ parameter with MACS [16] and visualized with Integrative Genomics Viewer (IGV) [17]. We counted the H3K4me3 peaks for promoter, exon and gene body respectively. We identified the H3K4me3-gained genes by comparing H3K4me3 peaks at promoter, in exon and gene body between control and GBM. If a gene that does not have a peak at promoter or in exon or in gene body in control gains at least one peak in GBM, we defined it as an H3K4me3-gained gene. Reversely, An H3K4me3-lost gene means that the gene has at least one peak at promoter or in exon or in gene body in control but loses the peak in GBM.

### Expression data of lower grade glioma and GBM from database TCGA

We retrieved the ‘level 3’ expression dataset of 27 lower grade glioma cases and 548 GBM cases from The Cancer Genome Atlas (TCGA) database (https://tcga-data.nci.nih.gov/tcga/tcgaHome2.jsp). Without changing the units of the data, we calculated the average expression level for each human gene in the lower grade glioma and in the GBM cases. The average expression data are used to test the correlation between H3K4me3 level and expression. Considering that H3K4me3 is enriched at promoters, we chose a TSS-vicinity region [–0.5k bp ∼ +1k bp] to calculate H3K4me3 reads counts and linked them to the expression. We also compared the expression differences between homeobox genes and cadherin genes. Based on the TFBSs data, we identified the target genes of transcription factors HOXA3, 5 and 9, and compared the expression of the target genes with the average expression data.

### Identifications of the genes with an amplified copy number

Although H3K4me3 ChIP-seq data are not appropriately used to genome-widely identify copy number variation (CVN), an abnormal ChIP-Seq reads enrichment can serve as a hint for an increased copy number at specific region. We searched the genes with an amplified copy number using the strategy in literature [18]. A genomic region that satisfies five criterions, (1) more than 1k bp, (2) the reads count in the region is 5-fold higher than the average reads count in GBM; (3) the reads count in the region is a 5-fold higher in GBM than in control; (4) the reads count in the region is 2-fold higher than average reads count in control and (5) excluding the possibility of repeating reads by checking reads sequences, is identified as an amplified region. *P*-value is calculated using the reads count of control as lambda (*λ*) and that of GBM as *k* in Poisson distribution (
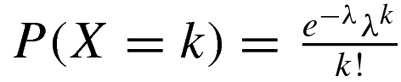
). A gene with an amplified region is called an amplified gene.

### Network construction

We investigated the interaction of homeobox genes (proteins) with STRING (v10) [19]. Firstly, the homeobox genes were inputted as ‘seeds’ to generate a primary network. Secondly, the primary network was expanded by clicking ‘+’ two times.

### Enrichment analysis

Enrichment analysis was carried out for KEGG pathway, gene ontology (GO) term, protein sequence feature and protein domain keywords using the functional annotation table module of DAVID (http://david.abcc.ncifcrf.gov/).

## RESULTS

### H3K4me3 levels decrease in GBM compared with GBM-surrounding tissue (control)

**Figure 1:**
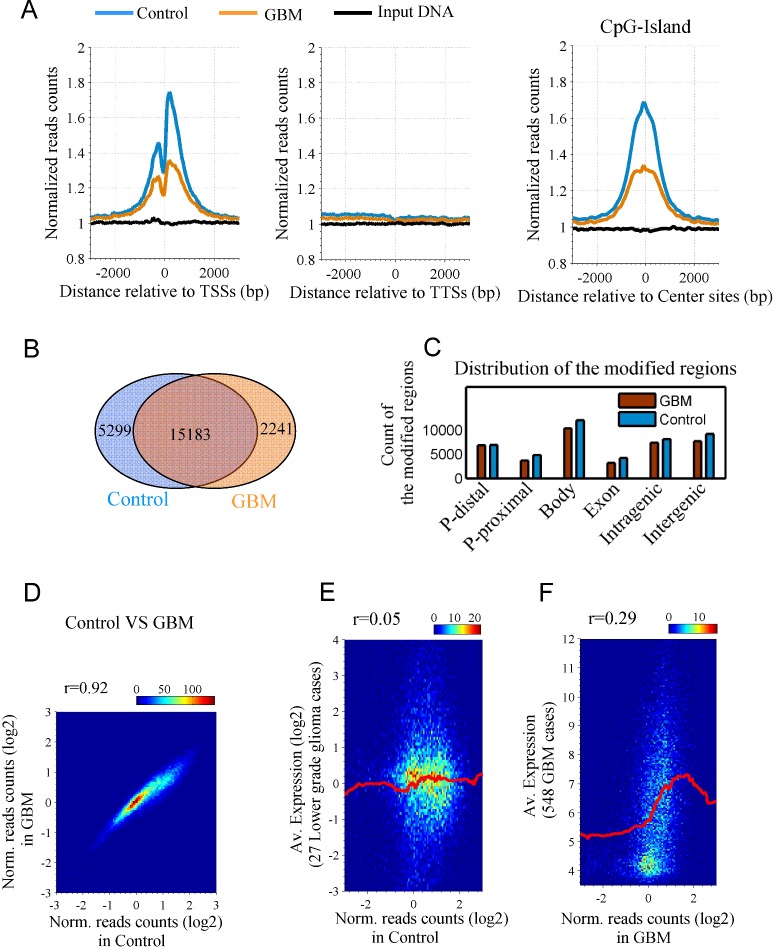
H3K4me3 decreases in GBM and positively correlated to the average expression of TCGA GBM cases (**A**) Profiles of H3K4me3 around TSSs (left panel), TTSs (mid panel) and centre sites of CpG-islands (right panel) in GBM and control (GBM-surrounding tissues) respectively. The profiles indicate normalized counts of mapped reads at each genomic locus. The normalization is calculated by dividing the mapped-reads counts at the locus with the total mapped-reads number. (**B**) Venn diagram indicates the overlapping number of MACS-called modification regions in control and GBM. (**C**) Distributions of the MACS-called peaks, P-distal,–5k bp ∼ +1k bp relative to TSSs; P-proximal,–250 bp ∼ +250 bp relative to TSSs. (**D**) A density dot plot comparison of the normalized reads counts in a TSS-vicinity genomic region [–0.5k bp ∼ +1k bp] in control (*x* axis) and GBM (*y* axis). Pearson's correlation coefficient (*r*)=0.92. (**E**) The normalized reads counts in the regions [–0.5k bp ∼ +1k bp] in control against to the average expression of the 27 lower grade glioma cases from TCGA database. Red line shows the smoothed curve for the expression data. (**F**) The same as in E except that the normalized reads counts is in GBM and the average expression data are of 548 GBM cases.

We examined H3K4me3 profiles around specific genomic sites (Figure 1A). In both GBM and control tissues, H3K4me3 was enriched around TSSs (Figure 1A), showing a typical distribution pattern of H3K4me3 [6]. The modification is also enriched at CpG islands (Figure 1A). In contrast, near the 3′ end of genes, we did not observe any enrichment of H3K4me3 (Figure 1A). The profiles of input DNA are low and flat without any pattern (Figure 1A), indicating successful ChIP.

Importantly, we found that H3K4me3 levels decrease at promoters and CpG islands in GBM compared with the control (Figure 1A), which is in agreement with previous studies [1,7]. We identified the H3K4me3 peaks with tool MACS, resulting 17424 and 20482 peaks for GBM and control respectively, with a decrease in 3058 in GBM (Figure 1B). This decrease occurs at promoter and in exon, intron and gene body (Figure 1C and Supplementary Figure S2A). Interestingly, the H3K4me3 peaks that are gained and lost in GBM are shorter than the average width of all the peaks (Supplementary Figure S2B), indicating that H3K4me3 alteration mainly occurs on small genomic regions. In literature, widespread shortening of broad H3K4me3 peaks in cancers is associated with repression of tumour suppressors [20].

We also noticed that more than 80% of the peaks do not change between control and GBM (Supplementary Figure S2C). The correlation of normalized reads counts between control and GBM is beyond 0.9 in a TSS-vicinity region [–0.5k bp ∼ +1k bp] (Figure 1D), indicating a similarity between GBM and GBM-surrounding tissues.

Since H3K4me3 associates to transcriptional activation, we calculated the correlation between H3K4me3 level and gene expression. So we estimated the average expression for human genes in the 27 lower grade glioma and 548 GBM cases from TCGA database and plotted the average expression against the H3K4me3 reads counts in the TSS-vicinity region [–0.5k bp ∼ +1k bp] (Figures 1E and 1F). The results indicated that the H3K4me3 is more correlated to the expression in GBM than in control.

Taken together, during the shift from GBM-surrounding tissue to GBM tissue, H3K4me3 decreases and positively correlates to the average expression of TCGA GBM cases.

### Homeobox genes gain H3K4me3 peaks and cadherin genes lose H3K4me3 peaks in GBM

**Figure 2:**
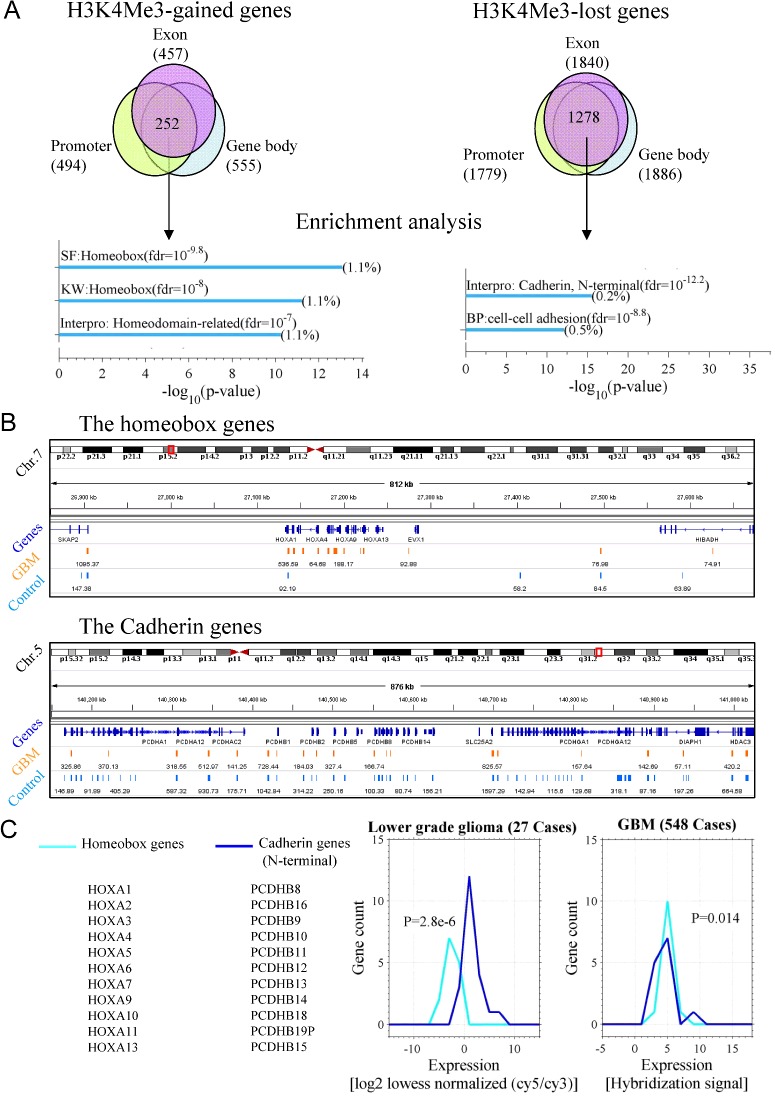
Homeobox genes gain H3K4me3 whereas cadherin genes lose the modification in GBM (**A**) H3K4me3 -gained and -lost genes and their enrichment analysis. Venn diagrams indicate the overlapped numbers of the genes that gain and lose H3K4me3. At first, the H3K4me3 -gained or -lost genes were identified at promoter, in exon and in gene body respectively (Supplementary Figure S3A). Then, the common genes were counted. Finally, an enrichment analysis was carried out for the 252 H3K4me3-gained genes and the 1272 H3K4me3-lost genes using the bioinformatics a tool DAVID. The false discovery ratio (fdr) and percentage of enriched genes to total genes are indicated. CC: cellular components; SF: PIR sequence feature; KW, PIR keywords; Interpro, protein domain. (**B**) H3K4me3 peaks near the homeobox genes (top) and the cadherin genes (bottom). (**C**) Expression of the homeobox genes and the cadherin genes in 27 lower grade glioma and 548 GBM cases.

We further identified the genes that gain and lose H3K4me3 peaks in GBM compared with control and resulted 252 H3K4me3-gained genes and 1278 H3K4me3-lost genes (Figure 2A and Supplementary Figure S3A). Enrichment analysis suggested that the 252 H3K4me3-gained genes are enriched in ‘homeobox’ term and the 1278 H3K4me3-lost genes are enriched in terms of ‘glycoprotein’, ‘intrinsic to membrane’, ‘signal peptide’ and ‘cadherin’ (Figure 2A and Supplementary Figure S3B). Figure 2(B) shows the details of H3K4me3 peaks for the homeobox and the cadherin genes. At the locus of the homeobox genes (from HOXA2 to HOXA13), H3K4me3 peak is present in control but absent from GBM (top panel in Figure 2B). In contrast, the H3K4me3 peak drastically reduces near the cadherin genes (from PCDHB8 to PCDHB15) from control to GBM (bottom panel in Figure 2B). H3K4me3 profiles of the genes indicate that the alteration occurs not only at promoter but in gene body (Supplementary Figure S4). We further validated the results in an independent GBM sample of literature (GSM ID: 1866131) [21]. The literature GBM data show more matched H3K4me3 peaks with the present GBM than that with the present control (Supplementary Figure S5A). In the literature GBM sample, we also found there are five H3K4me3 peaks around the homeobox genes (Supplementary Figure S5B, top panel), showing a slight increase in H3K4me3 in comparing with the present control. Around the cadherin genes, the literature data show very few peaks, indicating a loss of H3K4me3 in the GBM. The results suggested that the finding in the present GBM sample is reasonable at least in the literature data.

The homeobox genes contain a particular DNA sequence that encodes the homeodomain. The products of the homeobox genes play key roles in regulating development [11]. They often appear in clusters [22]. The cadherin-related genes are responsible for cell–cell adhesion. They belong to protocadherin beta gene cluster and also appear in a cluster on chromosome five [23].

Another gene group that lost the H3K4me3 modifications is enriched in GO term of ‘plasma membrane’ (Supplementary Figure S3B). Literatures suggested that the plasma membrane-related genes are in EGFR-associated signalling and cell ‘movement’ in GBM [24–28].

In short, our results suggest that H3K4me3 is lost in the cadherin genes and is gained in the homeobox genes.

Next, we investigated the expression of the homeobox genes and the cadherin genes. We estimated the average expression of the two groups of genes in two glioma types, the lower grade glioma and GBM cases, respectively. The distributions of the average expression are shown in Supplementary Figure S6. Here, we hypothesized the GBM-surrounding tissue, as a control in the present study, is comparable to the lower grade glioma. Providing that H3K4me3 is a gene activation mark [6,20], the two groups of genes will have a differential expression because they have different level of H3K4me3. We found that in the lower grade glioma (27 cases) the homeobox genes show a significantly lower expression than the cadherin genes (left panel in Figure 2C). However, in GBM (548 cases), the expression of the two genes groups is almost comparable (right panel in Figure 2C). This at least suggests that the expression of the homeobox genes is increased and the expression of the cadherin genes is decreased or either of them was true during the shift from GBM-surrounding tissue to GBM tissue. This is in line with the finding that HOX genes are up-regulated in GBM [28]. Also, we used tool ‘GEO profiles’ of NCBI to search the expression of two homeobox genes (HOXA5 and HOXA10) and two cadherin genes (PCDHA1 and PCDHB13) in different grades of the glioblastoma. From the low grade to the high grade, the expression of HOXA5 and HOXA10 increases whereas the expression of PCDHA1 and PCDHB13 decreases (Supplementary Figure S7). The result is in line with the present finding.

### A network suggests the alteration of H3K4me3 in homeobox genes associates with activation of cancer-related pathways

**Figure 3:**
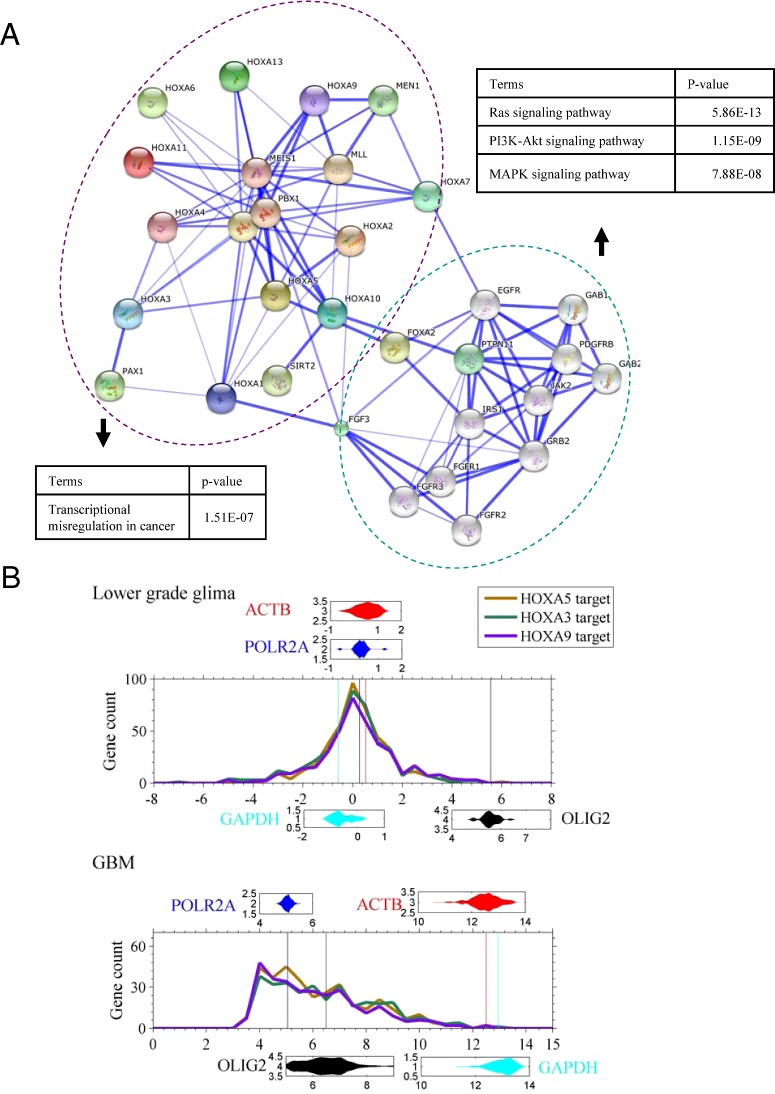
Homeobox genes connect important pathways of cancer cell (**A**) An interaction network constructed using the homeobox genes as ‘seeds’ with tool STRING. Line thickness relates to ‘confidence’ (combined score). The text shows the enriched terms by the genes in the network. (**B**) Expression distributions of the genes with conserved binding sites of HOXA3, HOXA5 and HOXA9 in both the lower grade glioma and the GBM of database TCGA. The data of the binding sites are retrieved from database USCS (https://genome.ucsc.edu/). The expression is averaged in the 27 lower grade glioma and the 548 GBM cases respectively. The insets show the violin plotting for the expression distributions of the genes GAPDH, ACTB, POLR2A and OLIG2 in the 27 lower grade glioma and the 548 GBM cases. The vertical lines indicated the average expression of the four genes.

Furthermore, we built a network for the homeobox proteins with tool STRING (see Methods). The network indicates that the homeobox proteins connect to the pathways of Ras-signalling, PI3K-Akt signalling and MAPK signalling (Figure 3A). The pathways are frequently activated in cancers [30]. If the H3K4me3-modified homeobox genes are activated in GBM, their products will subsequently activate the cancer-related pathways. In literature [13], MLL was thought to directly activate HOXA10, in turn, activate the tumorigenesis-related genes. We did observe a link from MLL to HOXA10 then to PTPN11 (Figure 3A). Thus, we inferred that the activation of HOXA10 by MLL is through the gain of H3K4me3 on HOXA10. Other important links include HOXA7-to-EGFR, HOXA1-to-FEG3 and HOXA10-to-FOXA2. We also noticed the links among the homeobox genes.

If the homeobox genes are activated due to the gain of H3K4me3 in GBM, transcription of their target genes should be promoted. We tested the hypothesis by comparing the average expression of the target genes of HOXA3, HOXA5 and HOXA9. In the comparison, the expression level of OLIG2 was used as a reference because OLIG2 is a clinical immunohistochemical marker for GBM and is highly expressed in GBM [1,31,32]. As expected, the target genes have a lower expression than OLIG2 in the lower grade glioma cases, whereas a higher expression than OLIG2 in the GBM cases (Figure 3B).

### Classification of the GBM subgroup

GBM has considerable heterogeneity in terms of genetics, epigenetics and the expression levels of particular genes [1]. Finally, we intended to classify the subgroup of this GBM case based on the ChIP-Seq data.

**Figure 4:**
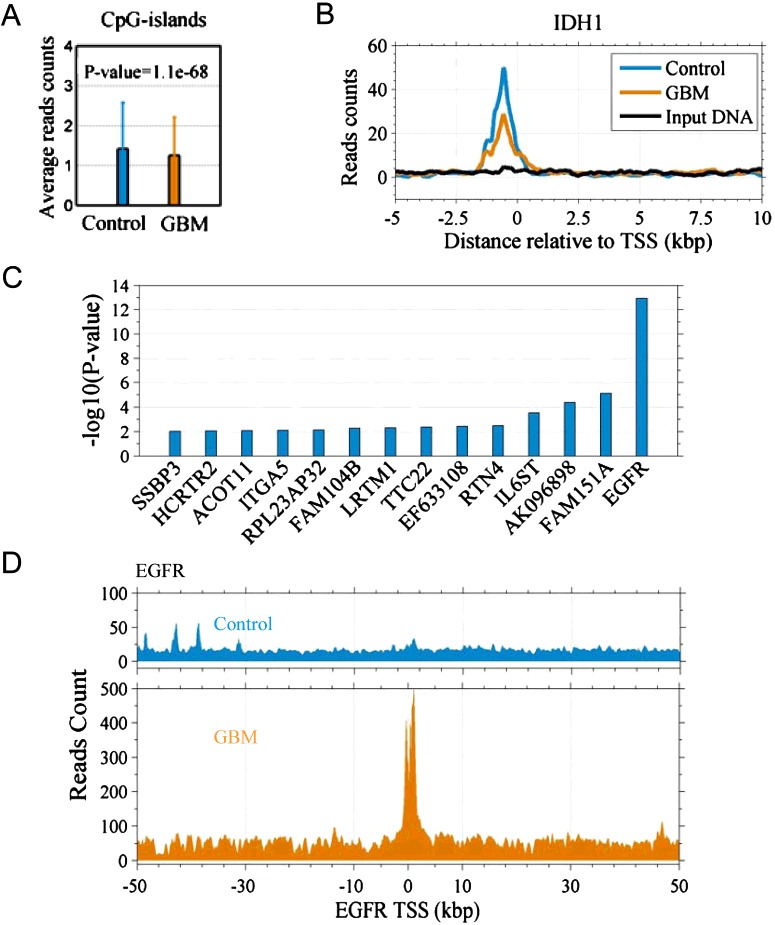
Inference of the subgroup of this GBM case (**A**) Two-sample *t*-test for the average read counts of 0.5-kbp region around CpG-islands between control and the GBM. (**B**) Profiles (reads counts) around TSS of gene IDH1. (**C**) The genes with an amplified copy number identified with H3K4me3 ChIP-Seq data. (**D**) Reads count of EGFR gene, the count is not normalized.

Considering that H3K4me3 is anti-correlated to DNA methylation in GBM [4,7], we believed that the DNA methylation level was increased because the level of H3K4me3 was decreased in this case of GBM (Figure 1), especially at CpG islands (*P*=1.1×10^−68^, two-sample *t*-test) (Figure 4A). According to a previous study [1], among the six GBM subgroups, H3F3A (K27), H3F3A (G34) and RTK I subgroups exhibit low DNA methylation. Thus, we inferred that the GBM case was not in these three subgroups.

In the IDH subgroup, the mutant IDH1 gene shows high expression [1]. Expression is positively correlated to enrichment of H3K4me3 at promoter [33]. However, we found that the H3K4me3 level was lower at the IDH1 gene promoter in GBM than in GBM-surrounding tissue (first panel in Figure 4B), suggesting that IDH1 expression is lower in GBM than in GBM-surrounding tissue, thus we exclude the IDH subgroup.

We called the genes that probably associate amplified copy number regions (see Methods) (Figure 4C). Gene EGFR shows a great reads coverage in GBM (*P*<10^−13^) (Figure 4D). We excluded the possibility of clone reads by checking reads sequences. It is likely that the EGFR genomic region is amplified due to an increase in copy number in GBM.

Based on the evidences above, we inferred that this case of GBM was probably in the RTK II subgroup, which is featured with DNA hypermethylation, OLIG2 high expression and EGFR amplification.

## DISCUSSION

We examined the distribution of H3K4me3 in a case of GBM and the GBM-surrounding tissues. Our finding suggested an H3K4me3 reduction at promoters and CpG islands in GBM compared with the surrounding tissues although there are approximately 80% of H3K4me3 peaks that mark at same loci in both tissues (Figure 1 and Supplementary Figure S3). Because of the epigenetic heterogeneity of GBM [1,3,4,8], the H3K4me3 reduction cannot be simply as a marker of GBM. Only a small correlation coefficient 0.29 exists between the H3K4me3 level and the average expression of TCGA GBM cases (Figure 1F).

Importantly, the genomic loci where H3K4me3 is greatly altered contain the homeobox genes and the cadherin genes (Figure 2). The homeobox genes and the cadherin genes respectively gain and lose the modification in GBM. HOXA10 gene can be activated by MLL in tumorigenesis [13]. We concluded this activation is associated with H3K4me3 gain in GBM. Moreover, the other homeobox genes, from HOXA2 to HOXA13, are methylated on H3K4 in GBM. These genes are all in connection with cancer pathways. Since the homeobox genes are in ‘glioma stem cell’ or ‘self-renewal’ phenotype in treatment resistance of glioblastoma [13,28,29]. Therefore, the H3K4me3-gained genes closely associated cell division and differentiation in GBM. On the other hand, the cadherin genes function in cell–cell adhesion. The loss of H3K4me3 on the genes will probably cause an inactivation of them, thus lead to invasive migration [34].

Literatures suggested that homeobox genes and cadherin genes are also in brain metastases [35,36]. Down-regulation of E-cadherin was observed in brain metastases from lung cancer samples [35]. In brain metastases from melanoma, DNA methylation was altered and the significantly affected genes were the homeobox family members, especially for HOXD9 [36]. At the present study, we did not know whether there is a link between the H3K4me3 gain on homeobox genes and the loss on the cadherin genes. It should be mentioned that all of the analysis is limited in the case although the main conclusion of the work can be inferred from the literatures.

## Abbreviations

ChIP-Seq: chromatin immunoprecipitation sequencing
EGFR: epidermal growth factor receptor
GBM: glioblastoma multiforme
GO: gene ontology
H3K4me3: tri-methylation on lysine 4 of histone H3
IDH: isocitrate dehydrogenase
TCGA: The Cancer Genome Atlas
TSS: transcription start site
TTS: transcription termination site
